# Calcium Deregulation: Novel Insights to Understand Friedreich’s Ataxia Pathophysiology

**DOI:** 10.3389/fncel.2018.00264

**Published:** 2018-10-02

**Authors:** Rosella Abeti, Alexander F. Brown, Marta Maiolino, Sandip Patel, Paola Giunti

**Affiliations:** ^1^Ataxia Centre, Department of Clinical and Movement Neurosciences, Institute of Neurology, University College London, London, United Kingdom; ^2^Department of Biomedical Sciences and Public Health, School of Medicine, Università Politecnica delle Marche, Ancona, Italy; ^3^Department of Cell and Developmental Biology, Division of Biosciences, University College London, London, United Kingdom

**Keywords:** FRDA, CGNs, cardiomyocytes, calcium, oxidative stress, sarcoplasmic reticulum, ryanodine receptors and mitochondrial membrane potential

## Abstract

Friedreich’s Ataxia (FRDA) is a neurodegenerative disorder, characterized by degeneration of dorsal root ganglia, cerebellum and cardiomyopathy. Heart failure is one of the most common causes of death for FRDA patients. Deficiency of frataxin, a small mitochondrial protein, is responsible for all clinical and morphological manifestations of FRDA. The focus of our study was to investigate the unexplored Ca^2+^ homeostasis in cerebellar granule neurons (CGNs) and in cardiomyocytes of FRDA cellular models to understand the pathogenesis of degeneration. Ca^2+^ homeostasis in neurons and cardiomyocytes is not only crucial for the cellular wellbeing but more importantly to generate action potential in both neurons and cardiomyocytes. By challenging Ca^2+^ homeostasis in CGNs, and in adult and neonatal cardiomyocytes of FRDA models, we have assessed the impact of frataxin decrease on both neuronal and cardiac physiopathology. Interestingly, we have found that Ca^2+^ homeostasis is altered both cell types. CGNs showed a Ca^2+^ mishandling under depolarizing conditions and this was also reflected in the endoplasmic reticulum (ER) content. In cardiomyocytes we found that the sarcoplasmic reticulum (SR) Ca^2+^ content was pathologically reduced, and that mitochondrial Ca^2+^ uptake was impaired. This phenomenon is due to the excess of oxidative stress under FRDA like conditions and the consequent aberrant modulation of key players at the SR/ER and mitochondrial level that usually restore the Ca^2+^ homeostasis. Our findings demonstrate that in both neurons and cardiomyocytes the decreased Ca^2+^ level within the stores has a comparable detrimental impact in their physiology. In cardiomyocytes, we found that ryanodine receptors (RyRs) may be leaking and expel more Ca^2+^ out from the SR. At the same time mitochondrial uptake was altered and we found that Vitamin E can restore this defect. Moreover, Vitamin E protects from cell death induced by hypoxia-reperfusion injury, revealing novel properties of Vitamin E as potential therapeutic tool for FRDA cardiomyopathy.

## Introduction

Friedreich’s ataxia is caused by frataxin insufficiency leading to changes in iron metabolism that inhibits mitochondrial respiration and promotes reactive oxygen species (ROS) generation, causing mitochondrial dysfunction, oxidative stress and subsequent mitochondrial iron accumulation (Oudit et al., 2006). These effects result in selected neuronal atrophy, where the primary sites of pathology are the dorsal root ganglia (DRG) (Dyck et al., 1968), and a characteristic proliferation of the synaptic terminals in the dentate nucleus of the cerebellum (Koeppen et al., 2007). The neuronal degeneration in DRG seems to be caused by a dying-back phenomenon triggered by Ca^2+^ deregulation, including failure to buffer Ca^2+^ and defective store-operated calcium entry (SOCE) mechanism (Molla’ et al., 2017). Ca^2+^ mishandling has been closely associated to oxidative stress mediated cell death as Ca^2+^ chelators proved to be protective (Wong et al., 1999; Mincheva-Tasheva et al., 2014). CGNs are also subjected to degeneration, in which we previously showed increase of oxidative stress and mitochondrial dysfunction in a FRDA mouse model (Abeti et al., 2016). We have now investigated whether oxidative stress can be also linked to Ca^2+^ mishandling in CGNs.

In addition to the pathology of the central and peripheral nervous system, a hypertrophic cardiomyopathy is commonly seen at the beginning of the disease, and this progressive cardiomyopathy is considered one of the most common causes of death in FRDA (Pastore and Puccio, 2013). Iron-induced oxidative stress has been associated with altered Ca^2+^ homeostasis. In particular, it has been associated with reduced peak systolic Ca^2+^ levels, slowed rates of Ca^2+^ relaxation and elevated diastolic Ca^2+^ levels in diseases such as hereditary haemolytic anemia and other iron-overload conditions (Navarro et al., 2010; Rehsia and Dhalla, 2010), which can lead to arrhythmias and sudden death, both of which have also been seen to occur in FRDA. We therefore investigated whether the lack of frataxin in cardiomyocytes could be relevant to Ca^2+^ homeostasis, to this purpose we have employed two different cell lines of cardiomyocytes, one similar to adulthood and one neonatal and also primary cardiomyocytes from an FRDA mouse model. The usage of neonatal and adult cardiomyocytes allowed us to understand whether there could be any differences during cardiac development in FRDA pathophysiology. Deregulation of Ca^2+^ homeostasis has been previously observed in neuronal models of FRDA (Bolinches-Amoros et al., 2014; Mincheva-Tasheva et al., 2014), however, this is the first study focused on Ca^2+^ signaling in cardiomyocytes of FRDA models. Given the known association between iron-induced oxidative stress and altered Ca^2+^ homeostasis, as well as the overlap with some pathogenic aspects of FRDA and other neurodegenerative iron-overload diseases (Isaya, 2014), this study could be of great benefit in elucidating the pathophysiological mechanism of FRDA in neuronal and heart cells. The sarcoplasmic reticulum (SR) is one of the major Ca^2+^ stores in cardiomyocytes. Ca^2+^ influx occurs through ryanodine receptors (RyRs Type 2) operating under a system of Ca^2+^-induced Ca^2+^ release (CICR), where the presence of Ca^2+^ itself induces further Ca^2+^ release from SR stores, generating a Ca^2+^ wave that contributes to cardiac action potential (Puccio et al., 2001). Thus, Ca^2+^ has a vital role in excitation-contraction coupling in cardiomyocytes. RyRs together with SR Ca^2+^ ATPase (SERCA) pumps contain many free cysteine (Cys) residues that are sensitive to oxidation (Stephanou et al., 1998; Ying et al., 2008). Oxidative stress may causes potentiation to RyRs increasing the probability and the duration of the open state of the RyR channel, which in turn leads to SR Ca^2+^ leak (Ying et al., 2008). We therefore investigated the activity of RyRs and the SR Ca^2+^ content to understand whether those could be affected in cardiac FRDA-like models. Oxidative stress has already been observed in several models of FRDA (Lamarche et al., 1980; Emond et al., 2000; Puccio and Koenig, 2000; Bradley et al., 2004; Abeti et al., 2016). Here, we confirm that oxidative stress is present in CGNs and cardiomyocytes with a reduced level of frataxin and this impairs Ca^2+^ homeostasis. To protect cells from oxidative stress we have employed Vitamin E, a natural lipid soluble antioxidant processed in cellular membranes and in particular in the mitochondria. Previous studies on Vitamin E combined with Coenzyme Q10 (CoQ10) on FRDA patients showed a beneficial effect on mitochondrial bioenergetic monitored directly in the cardiomyocytes and muscle cells with magnetic resonance spectroscopy (^31^P-MRS) (Lodi et al., 2001; Hart et al., 2005). At that time there was not enough natural history on FRDA patients and cardiomyopathy to have a definitive conclusion of efficacy (Cooper and Schapira, 2007). We observed the mitochondrial Ca^2+^ uptake which improved significantly in frataxin knocked down cardiomyocytes. Moreover, we have challenged the cells with hypoxia-reoxigenation (H/R) injury to understand how cells were coping during H/R damage. The beneficial effect of Vitamin E improved cell viability in cardiomyocytes stressed with H/R challenge. Our results confirm that Vitamin E has a protective effect on FRDA like cardiomyocytes. Therefore, this compound could be proposed as a therapy for FRDA patients.

## Materials and Methods

### Primary Cultures

Cerebellar granule neurons were obtained from YG8R mice and their matching control Y47R. YG8R is a humanized mouse model, which contains a human FXN YAC with 190+82 GAA repeats on a mouse Fxn null background, that recapitulates the progressive disease phenotype shown in humans (Al-Mahdawi et al., 2006; De et al., 2007; Abeti et al., 2016), while Y47R (Control) has 9 GAA repeats over the same background (Jackson Laboratory; this study was carried out in accordance with the recommendations of Animal (Scientific Procedures) Act 1986 (ASPA). The protocol was approved by the UCL Animal Welfare and Ethical Review Body (AWERB). CGNs were isolated with the method described in Abeti et al. (2015, 2016) with few modifications. In brief, cerebella were isolated and homogenized and dissociated with 0.25% trypsin-EDTA (Sigma Aldrich). CGNs were plated on, pre-coated with Poly-D-lysine (1 mg/ml), glass coverslips. Cells were maintained in Neurobasal with 2% of B27 (Invitrogen) and 2 mM L-glutamine, 1% of penicillin/streptomycin (Sigma Aldrich) and 25 mM KCL (to keep the CGNs slightly depolarised (Ramay et al., 2010)). To avoid glial proliferation 10 μM AraC was added after 24 h from the plating. CGNs were used 7 days after plating. Cells were maintained in a humidified incubator at 37°C and at 5% CO_2_.

Primary cultures of neonatal cardiomyocytes were obtained from 6 day-old YG8R and Y47R mice (Al-Mahdawi et al., 2006; De et al., 2007; Abeti et al., 2016). Cardiomyocytes were prepared with the method described by Stephanou et al., (Schwartz et al., 2002). Briefly hearts were homogenized and digested with isolation buffer solution composed (millimolars): 116 NaCl, 20 HEPES, 0.77 NaH_2_PO, 5.5 D-Glucose, 5.4 KCl, 0.4 MgSO_4_, pH adjusted to 7.35. In 100 mL isolation buffer were added: 60 mg collagenase type II CLS2 (∼250 μ/mg, Worthington), 25 mg pancreatin from porcine pancreas (Sigma). After digestion and cardiomyocytes extraction process the cells were plated in Dulbecco’s Modified Eagles Medium supplemented with 15% fetal calf serum and 1% peniccilin /streptomicyn, and maintained in incubator at 37°C, 5% CO_2_.

### Cell Cultures

HL-1 (murine adult cells; a generous donation from the lab of Professor Sean Davidson, Hatter Institute UCL) and H9c2 (rat neonatal cells; ATCC) were cultured. HL-1 cells were maintained in Claycomb media (Claycomb et al., 1998) with 10% Fetal Bovine Serum (FBS; Invitrogen) 100 U/ml penicillin (Sigma Aldrich), 100 μg/ml streptomycin (Sigma Aldrich), 0.1 mM norepinephrine and 2 mM L-glutamine. Prior plating flasks were coated with a solution containing 0.02% gelatin and 5 μg/ml fibronectin. H9c2 cells were maintained in DMEM with 10% FBS and 2 mM L-glutamine. Cells were harvested with 0.25% trypsin-EDTA (Sigma Aldrich), then inactivated with complete media and plated on 25 mm glass coverslips or directly into a 6 multi-wells plate. All cell lines were maintained in a humidified incubator at 37°C and at 5% CO_2_.

### Transfection

To obtain frataxin knock down (FxnKD) cells were tranfected with Lipofectamine 2000, accordingly to the manufacturer instruction. Fxn-siRNA was co transfected with YFP-tagged plasmid (pcDNA3-YFP). Mm_Fxn_1 FlexiTube siRNA was used as the predesigned siRNA against mouse frataxin (SI01007139, Qiagen) in HL-1 cells and Rat_Fxn FlexiTube siRNA (SI02902557, Qiagen) was used in H9c2 cells. Negative control (Scr) was achieved with AllStars Negative Control siRNA (1027418, Qiagen) and Rat_Fxn FlexiTube siRNA was used in H9c2 cells.

### Imaging: ROS Measurements

Cells were loaded with dihydroethidium (Het; 5 μM; Thermo scientific) in HBSS (Sigma), which measures superoxide in the cytosol. This fluorophore was either used ratiometrically by exciting the unoxidised form in blue (364 nm) and the oxidized form in red (543 nm) or by measuring only the oxidized form. The ratio (543/364 nm) was applied for each cell measurement. Rates were then calculated. Mitochondrial ROS were measured with 1 μM CM-H2Xros (Thermo scientific) loaded for 20 min prior the beginning of the experiments.

### Lipid Peroxidation Measurements

To measure lipid peroxidation, cells were loaded with 10 μM C11 BODIPY (581/591) for 10 min prior the beginning of the experiments. The dye was excited at 488 nm (oxidized form) and 561 nm (un-oxidized form) (Al-Mahdawi et al., 2006; Abeti et al., 2015), detected with 710 Zeiss Confocal VIS CLSM equipped with a META detection system and a 40x oil immersion objective. The ratio of 488/561 nm was analyzed and the rates were then calculated in ArbU/minutes.

### Calcium Measurements

Transfected cells were loaded with 5 μM Fura-2-AM (340/380 nm) or 5 μM Fluo4-AM (488 nm) to measure cytosolic Ca^2+^ and 5 μM X-Rhod1 AM (562 nm) to measure mitochondrial Ca^2+^ levels, in HBSS (Sigma). Dyes were incubated for 20 min and washed three times prior experiments. For all the experiments with Thapsigargin HBSS was removed prior the beginning of the recordings and replaced with a Ca^2+^ free saline. Fluorescence measurements were obtained either using a cooled charge-coupled device (CCD) camera (Hamamatsu, Orca ER) or 710 Zeiss Confocal system.

### Measurement of ΔΨ_m_

Tetramethyl rhodamine, methyl ester (TMRM) is a cationic, cell-permeant fluorescent dye readily sequestered by healthy mitochondria. To measure small differences we have used a quenching mode. Cells were incubated 20 min with 500 nM TMRM and washed out. ΔΨ_m_ depolarization was detected as an increase in fluorescence (*de-quenching*). At the end of each experiment 1 μM Carbonyl cyanide-*4*-(trifluoromethoxy) phenylhydrazone (FCCP) was applied. FCCP is protonophore that dissipate the potential completely. TMRM was visualized using an excitation wavelength of 561 nm with a long pass filter using 710 Zeiss Confocal microscope.

### Immunofluorescence

To visualize the FxnKD we have used immunofluorescence (Abeti et al., 2015). Briefly, cells were fixed with 4% PFA, permeabilised with 0.5% Triton-X and incubated over night with an anti-frataxin antibody [17A11] (Abcam, ab113691; 1 μg/ml), and detected with a secondary antibody Alexa Fluor 568 goat anti-mouse (Thermo Fisher Scientific), diluted 1:1000. Nuclei were detected loading the cells with 300 nM DAPI for 5 min. Images were obtained using a Zeiss 710 LSM equipped with a META detection system and a 40x oil immersion objective.

### Hypoxia/Reperfusion (H/R) and TUNEL Assay

After 48 h from transfection the culture media was replaced with HBSS (Sigma) without glucose and cells placed in a hypoxic chamber saturated with Argon for 1 h (Hypoxia). Afterward, 5.5 mM D-glucose was added and cells were kept in a humidified incubator at 37°C and at 5% CO_2_, for 2 h (Reoxigenation). 200 μM Vitamin E was added 30 minutes before the H/R treatment, in the samples indicated. Terminal deoxynucleotidyl transferase dUTP nick end labeling (TUNEL) assay was performed in order to detect apoptotic cell death accordingly to the manufacture instructions (Roche).

### Statistical Analysis

Statistical analysis was performed with Excel, Origin 9 (Microcal Software Inc.) and GraphPad5 software (GraphPad Software, La Jolla, CA, United States). Results are expressed as means ± SEM or SDM. Mann–Whitney *U* test and ANOVA tests were applied when appropriate and the point of minimum acceptable statistical significance was taken to be 0.05 with Bonferroni correction. Representative averages were taken from *n* > 3 independent experiments.

## Results

### Oxidative Stress in Neurons and Cardiomyocytes of FRDA Models

**FIGURE 1:**
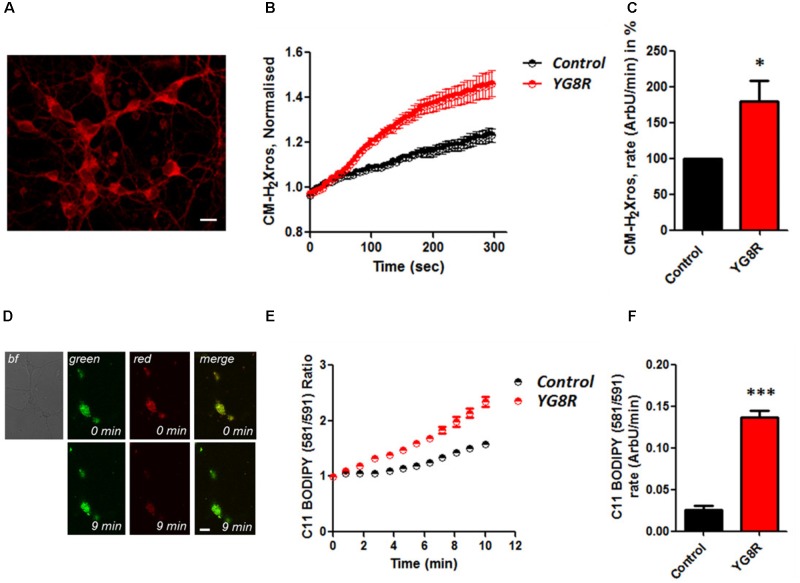
Oxidative stress in CGNs of FRDA mouse model. **(A)** The picture shows the loading of 1 μM CM-H_2_Xros in CGNs. **(B)** The graph shows the kinetic curves normalized, for Control (black) and YG8R (red). **(C)** The histogram represents the rates of ArbU over minutes in percentage normalized to the Control. mROS were significantly increased in YG8R CGNs compared to Control (^∗^*p* < 0.05). **(D)** The picture shows CGNs loaded with 10 μM C11 BODIPY (581/591), top left bright field (bf), at 0 minutes it is shown the oxidized form (top green), the un-oxidized form (top red) and the merge; at 9 min fluorescence it is shown the oxidized form increased (bottom green), the un-oxidized form decreased (bottom red) and the merge. **(E,F)** Show respectively the kinetic curves and the rates the lipid peroxidation (^∗∗∗^*p* < 0.0005). Scale bar (20 μm).

**FIGURE 2:**
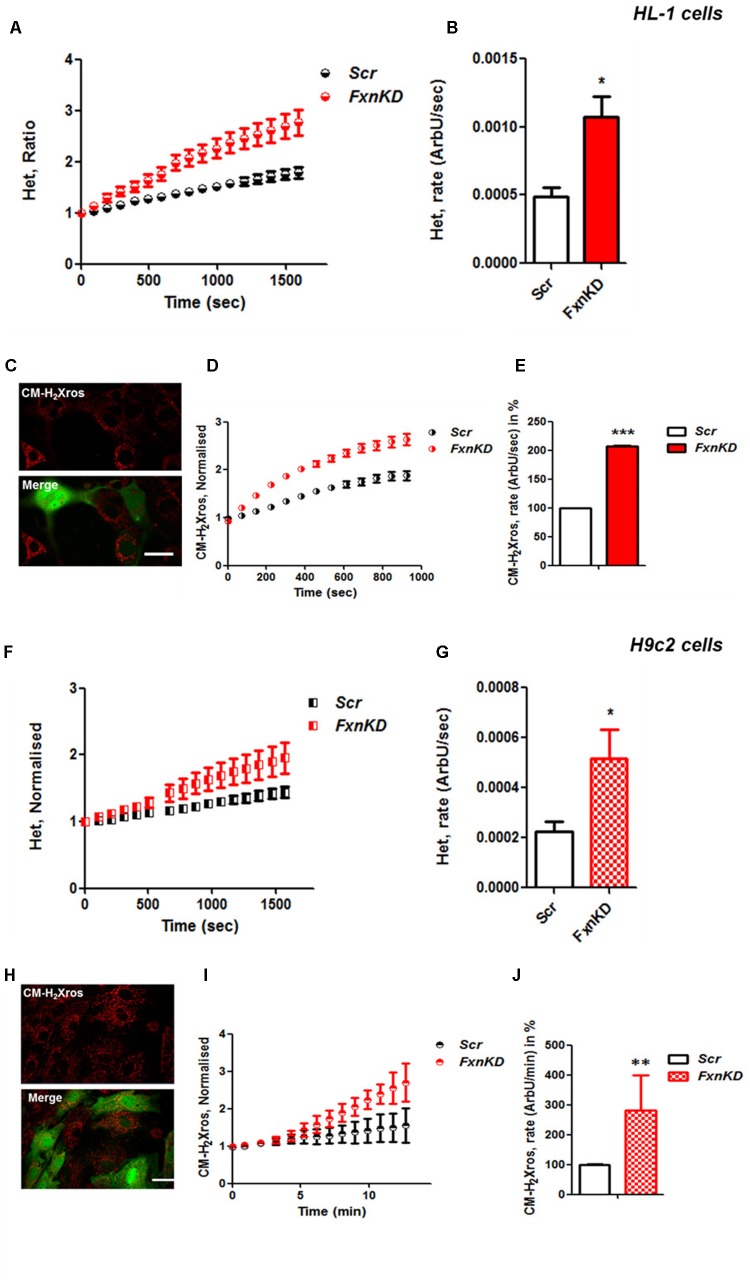
Frataxin decrease causes oxidative stress in cardiomyocytes. **(A,B)** HL-1 cells, Scr and FxnKD, were loaded with 10 μM dihydroethydium (Het) and imaged over time. **(A)** The kinetic curve show an increase of Het, meaning an increase of cytosolic ROS in FxnKD cells (red). **(B)** Confirmed by the rate of the dye (^∗^*p* < 0.05). **(C)** Here it is shown the loading of the dye (red) and the transfected cells (green in the merge). **(D,E)** The kinetic curves and the histogram represent the increase of mROS measured with 1 μM CM-H2Xros. Mitochondrial ROS were significantly increased in FxnKD (^∗∗∗^*p* < 0.0005). **(F,G)** We report the results of cytosolic ROS in H9c2 cells, that also showed a relevant increase in FxnKD (^∗^*p* < 0.05). **(H–J)** Here we report similar mROS measurements in H9c2 cells (^∗∗^*p* < 0.005). Scale bar (20 mm).

It is well established that frataxin decrease causes an elevation of ROS in many cell types (Pandolfo and Pastore, 2009; Abeti et al., 2015, 2016; Molla’ et al., 2017). Therefore we first confirmed that our models of study showed excessive oxidative stress. CGNs from YG8R mice (FRDA mouse model; see Materials and Methods) showed a significant increase in mitochondrial ROS (mROS) generation compared to control cells. **Figure 1** shows the loading of the dye (CM-H_2_Xros) in CGNs and the kinetic curves and rates generated by Control and YG8R neurons (**Figures 1A–C**; *YG8R* 180 ± 32; ^∗^*p* < 0.05). By measuring lipid peroxidation using C11 BODIPY (581/591), which fluoresces at two wavelengths (the green fluorescence increases upon oxidation the red decreases; **Figure 1D** illustrative figure at 0 min and at 9 min of the experiment) we performed the ratio and calculated the rates. This the presence of overall oxidative stress in this model, as YG8R neurons showed a significant increase in rate compared to control (**Figures 1E,F**; *Control* 0.025 ± 0.009, *YG8R* 0.137 ± 0.01; ^∗∗∗^*p* < 0.0005). For our study on cardiomyocytes we choose two cell lines, representative of both adult and juvenile phenotypes. HL-1 cells (murine adult cardiomyocytes) and H9c2 cells (rat neonatal cardiomyocytes) were co-transfected with an empty vector expressing YFP and scramble siRNA (Scr) (as control) or mFxn siRNA to achieve FxnKD. The efficiency of the FxnKD was assessed via immunocytochemistry and we found that the level of frataxin was significantly reduced when compared to control cells (**Supplementary Figures S1A–D**). To assess the oxidative stress in our cardiac models, we measured cytosolic and mROS. By loading the cells with dihydroethidium (Het), a fluorophore which becomes fluorescent in red upon oxidation, sensing ROS within the cell, we measured cytosolic ROS and found already a significant increase in resting condition when compared Scr to FxnKD, in HL-1 cells (**Figures 2A,B**). The curves in **Figure 2A** show the ratio of kinetic traces obtained from recording the blue (un-oxidized) and red (oxidized) fluorescence over time (see Materials and Methods). The rate of fluorescence increase calculated over time revealed a significant increment in FxnKD (**Figure 2B**; *Scr* 0.0005 ± 0.0002, *FxnKD* 0.001 ± 0.0008; ^∗^*p* < 0.05). We then detected mROS generation (**Figure 2C** shows the loading of the dye and the YFP transfected cells), using CM-H_2_Xros. Our data show that FxnKD cells have an increased level of mROS (**Figures 2D,E**; *FxnKD* 207.57 ± 0.79, ^∗∗∗^*p* < 0.0001). We have repeated the experiments on H9c2 cells (recording only the oxidative form of Het) and found similar results. **Figures 2F,G** show an increase of cytosolic ROS (**Figure 2G**; *FxnKD* 228.8 ± 2.65, ^∗^*p* < 0.05). Also mROS were increased in this model (**Figures 2H–J**, *FxnKD* 280.1 ± 118.5, ^∗∗^*p* < 0.005). These results demonstrate that by reducing the level of frataxin expression there is an overall increase of ROS both in the cytosol and the mitochondria. Having assessed the oxidative stress in our FRDA-like models we then wondered whether this could affect Ca^2+^ homeostasis.

### Calcium Mishandeling in FRDA-Like CGNs

**FIGURE 3:**
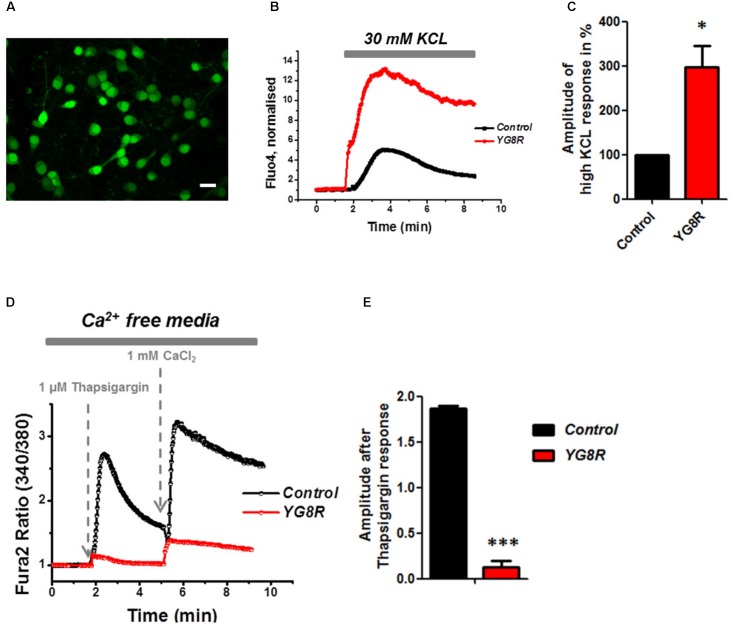
Failure to restore Ca^2+^ homeostasis after depolarization in YG8R CGNs. **(A–C)** CGNs were loaded with Fluo4-AM **(A)** and challenged with 30 mM KCL **(B)**, amplitudes were measured and showed a higher response in YG8R than Control (**C**; ^∗^*p* < 0.05). **(D,E)** CGNs response to 1 μM Thapsigargin and 1mM CaCl_2_, and relative amplitudes (^∗∗∗^*p* < 0.0005). Scale bar (20 μm).

Mitochondrial dysfunction and oxidative stress present in CGNs of FRDA-like model contribute to neuronal death (Abeti et al., 2016), and therefore potentially damaging other signaling pathways within the cells such as Ca^2+^ homeostasis. By challenging CGNs with a depolarizing injury we have assessed the Ca^2+^ response in control and YG8R. Cells were loaded with Fluo4-AM (**Figure 3A**) and challenged with 30 mM KCL (**Figure 3B**). The Ca^2+^ response to high KCL in YG8R CGNs was higher than the Control and more importantly there was no recovery to Ca^2+^ resting levels. The amplitude of this response was calculated in percentage to control and YG8R cells showed a significant difference (**Figure 3C**; *YG8R* 298 ± 96, ^∗^*p* < 0.05).

During depolarization Ca^2+^ enters the plasma membrane increasing the cytosolic levels but then it is quickly sequestered by the internal stores to restore the Ca^2+^ homeostasis and neurons go back to resting condition, ready for the next action potential. FRDA-like neurons showed an impairment on restore the resting state and a decreased level of ER Ca^2+^. Indeed, when YG8R cells were challenged with Thapsigargin the response was smaller (**Figures 3D,E**). The amplitude of the response was much lower in YG8R than the control (**Figure 3E**, *Control* 1.8 ± 0.02, *YG8R* 0.13 ± 0.07, ^∗∗∗^*p* < 0.0005) and also by adding Ca^2+^ there was a little response. These results could suggest that the sarcolemmal ATPase (SERCA) was impaired. Further investigation much be carried out to verify this possibility.

### Impaired Calcium Homeostasis in FRDA-Like Cardiomyocytes

**FIGURE 4:**
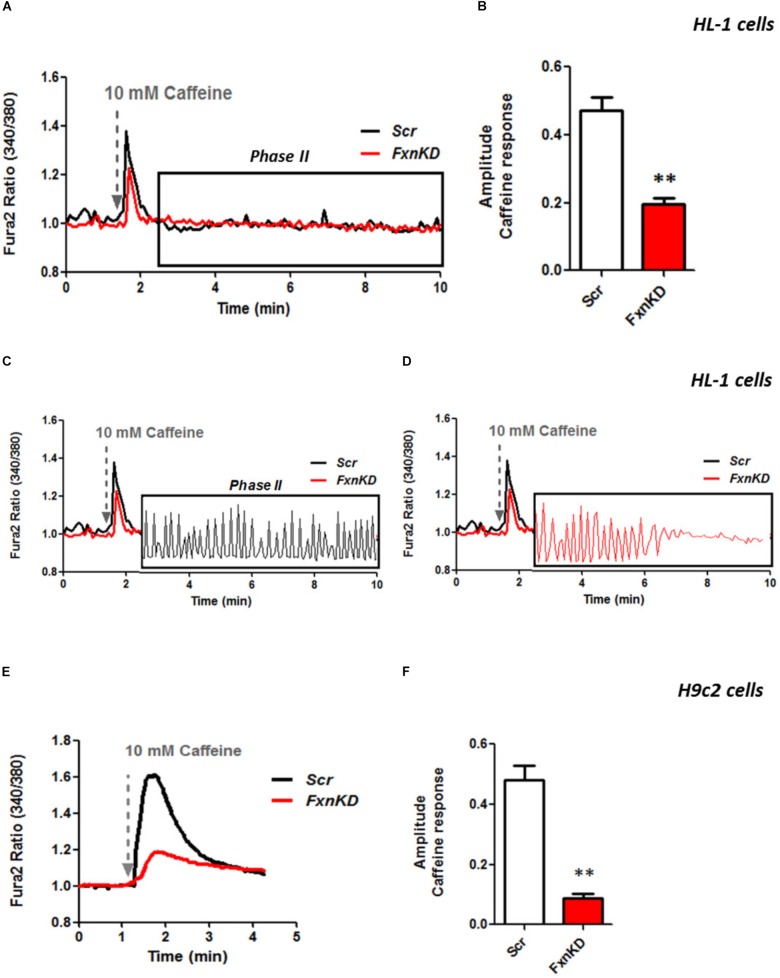
FxnKD induces a smaller caffeine response and RyRs potentiation. **(A,B)** HL-1 cells FxnKD shows a smaller caffeine response compared to Scr cells. **(A)** Shows the kinetic of an average of cells and **(B)** reports the full sets of experiments combined together showing the decreased amplitude in FxnKD cells (^∗∗^*p* < 0.005). **(C,D)** Highlight phase II of the Ca^2+^ response in Scr cells and in FxnKD cells. Showing an early end of the Ca^2+^ response probably ascribed to energy failure. **(E,F)** In H9c2 cells the diminished caffeine response in FxnKD was even more marked than HL-1 cells, however, the second phase of Ca^2+^ response was flat and the receptors saturated (^∗∗^*p* < 0.005).

**FIGURE 5:**
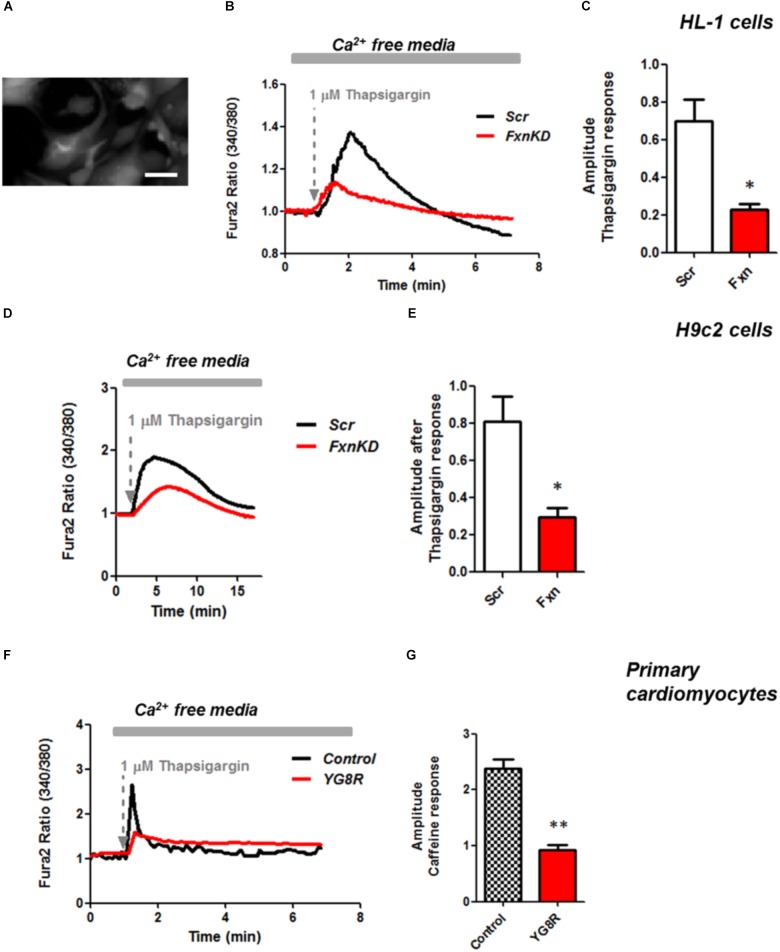
Frataxin silencing causes SR Ca^2+^ store depletion in cardiomyocytes. **(A)** Loading of Fura2-AM. **(B,D,F)** FxnKD cells show a marked difference to 1 μM thapsigargin response compared to Scr. **(C–G)** The amplitude of the response in relative values % shows a significant decrease in FxnKD cells which reveals a decrease of Ca^2+^ content in the SR (^∗^*p* < 0.05; ^∗∗^*p* < 0.005). Scale bar (20 mm).

It is well known that oxidative stress may cause alteration at many levels in Ca^2+^ homeostasis (Ermak and Davies, 2002). In order to understand whether the detected oxidative stress could inflict aberrant modulation of Ca^2+^ signaling in cardiomyocytes of FRDA models, we challenged the cells with 10 mM caffeine, an agonist of RyRs which elicits Ca^2+^ from the SR. Any alteration in SR Ca^2+^ content or perhaps an inhibition or a potentiation of the RyRs could be reflected in the intracellular Ca^2+^ ([Ca^2+^]_i_) response. Cells were loaded with 5 μM Fura2-AM and images recorded every second. Interestingly the response to caffeine was remarkably smaller in FxnKD HL-1 cells compared to control (**Figures 4A,B**; *Control* 0.5 ± 0.09, *FxnKD* 0.2 ± 0.05, ^∗∗^*p* < 0.005). This result could indicate that RyRs were potentiated to extrude more Ca^2+^ than what was necessary to restore the Ca^2+^ homeostasis and/or that SERCA pumps were not efficiently pumping Ca^2+^ into the SR. By looking at the second phase of the Ca^2+^ response, in HL-1 cells, we observed that the CICR rhythmic signal was different in the Scr compared to FxnKD (**Figures 4C,D**). The frequency and the amplitude of the signal were higher in FxnKD than the controls implying a potentiation of the RyRs which then stabilizes above baseline, reflecting an energetic collapse of the system. At the same time the decreased amplitude of the caffeine response could underline a mild impairment at the level of SERCA pumps. Similarly, the caffeine response was reduced in transfected H9c2 cells (**Figures 4E,F**; *Control* 0.5 ± 0.1, *FxnKD* 0.09 ± 0.04); however, there were no signs of oscillatory events, perhaps due to the different developmental stage. The detrimental effect of Ca^2+^ signaling could be explained by the exacerbation of the oxidative stress in the FRDA like cells, as RyRs and SERCA have cysteines that are affected by the variation of the oxidative environment (Stephanou et al., 1998; Ying et al., 2008). Perhaps both or one of the two calcium transport systems could be altered. Moreover, a reduction in SR Ca^2+^ uptake under pathological heart conditions would be exacerbated by a leaky Ca^2+^ release from the SR. For example it is known that hyperphosphorylation of the RyR2s (the most prominent isoform in cardiac muscle) increases their probability of being open (Ying et al., 2008) and also the reduction of SERCA2a (the most prominent isoform in cardiac muscle) is strictly correlated to heart failure (Diaz et al., 2005). We have then tested the content of SR via Thapsigargin (1 μM) administration, to estimate the level of Ca^2+^ in the SR. The [Ca^2+^]_i_ content elicited by the FxnKD cells was significantly reduced compared to the controls [**Figure 5A** loading of Fura 2AM. **Figure 5B**, Fura2 ratio kinetic curves; **Figure 5C**, *Control* 0.7 ± 0.2, *FxnKD* 0.2 ± 0.06, ^∗^*p* < 0.05). The decrease of [Ca^2+^]_i_ in response to Thapsigargin was also observed in H9c2 cells (**Figures 5D,E**; *Control* 0.8 ± 0.3, *FxnKD* 0.3 ± 0.1, ^∗^*p* < 0.05). To corroborate our data obtained on cell lines we monitored [Ca^2+^]_i_ also in primary cardiomyocytes of YG8R mice. The results showed yet again a significant decrease in Ca^2+^ content of the SR (**Figures 5E–G**, *Control* 2.3 ± 0.4, *YG8R* 0.9 ± 0.18, ^∗∗^*p* < 0.005).

**FIGURE 6:**
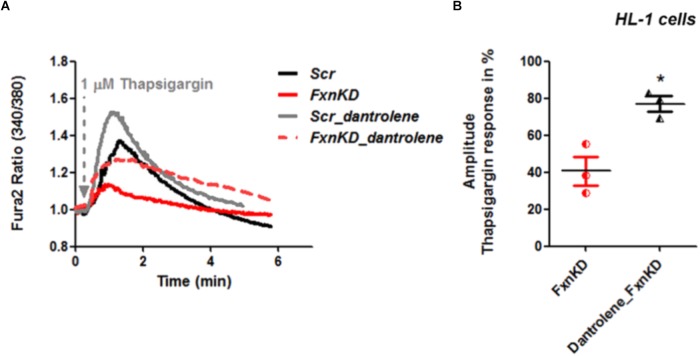
Dantrolene prevents SR Ca^2+^ store depletion in FxnKD cardiomyocytes. **(A)** HL-1 cells show an increased calcium response after pre-incubation with dantrolene, an inhibitor of RyRs. **(B)** Comparing the response between FxnKD and Dantrolene_FxnKD we found a significant increase of the thapsigargin–induced calcium response in dantrolene pre-treated FxnKD, demonstrating that the RyRs were not inhibited (^∗^*p* < 0.05).

By pre-treating cardiomyocytes with 20 μM dantrolene, an inhibitor of RyRs, we assessed whether the receptors were already inhibited or not. The results revealed that the response to Thapsigargin was higher in HL-1 cells pre-treated with dantrolene than without (**Figures 6A,B**). In particular by comparing FxnKD cells untreated and pre-treated with dantrolene we found a significant increase of [Ca^2+^]_i_ represented in the scatter plot showing the amplitude of Thapsigargin response (**Figure 6B**; *FxnKD* 40.7 ± 13.5, *Dantrolene_FxnKD* 77 ± 7, ^∗^*p* < 0.05). These results show that RyR2s were not inhibited and perhaps activated, causing the decrease of Ca^2+^ in the SR, which was restored with dantrolene. Potentiation of RyR2s causes an excessive release of Ca^2+^ from the SR.

### Mitochondrial Energy Imbalance Causes Pathophysiology in FRDA

**FIGURE 7:**
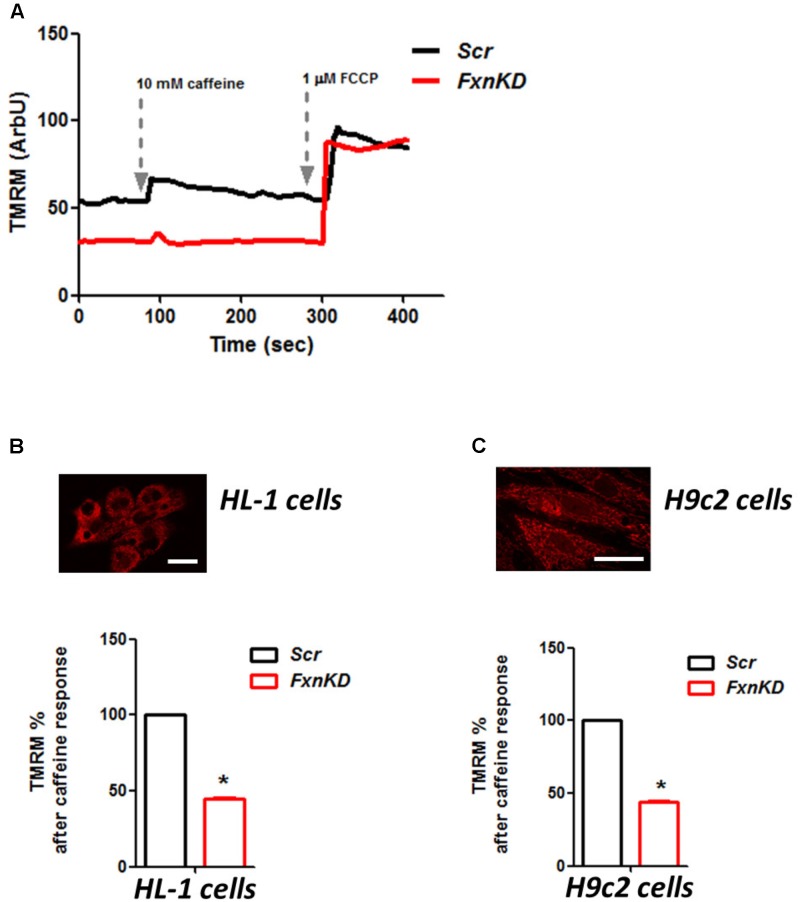
FxnKD causes an abnormal energetic response to caffeine in cardiomyocytes. **(A)** TMRM was loaded in de-quence mode, at high concentration, where a depolarization of ΔΨ_m_ is seen by an increase of fluorescence. The response to caffeine depolarises the potential as the mitochondria are active taking up Ca^2+^ to restore the Ca^2+^ homeostasis of the cell. **(B,C)** In FxnKD cells the response is reduced revealing that the mitochondria may not take up Ca^2+^ correctly (^∗^*p* < 0.05). Scale bar (20 μm).

To investigate the role of mitochondria in this process we looked at the mitochondrial membrane potential (ΔΨ_m_) during caffeine administration. We found that in both cell lines the response to caffeine was combined to a depolarization of ΔΨ_m_ in control cells and that was significantly reduced in FxnKD (**Figures 7A–C**; B, *FxnKD* 45.1 ± 0.4, C *FxnKD* 44 ± 0.36, ^∗^*p* < 0.05). This implied that instead of a physiological mitochondrial Ca^2+^ [Ca^2+^]_m_ load, which can happen when the cytosolic Ca^2+^ is low, we were experiencing perhaps a decreased Ca^2+^ uptake from the mitochondria.

**FIGURE 8:**
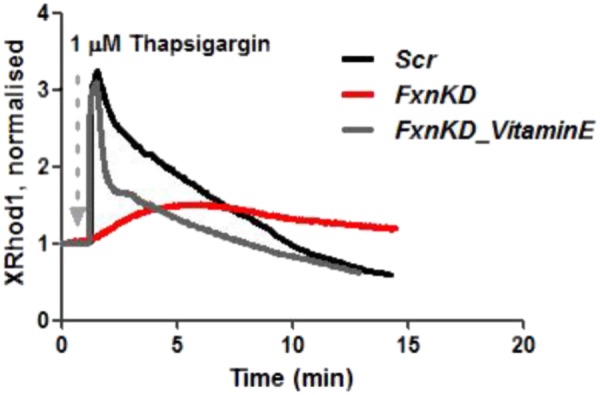
Mitochondrial Ca^2+^ is altered during Thapsigargin response. During Thapsigargin response mitochondrial Ca^2+^ uptake is reduced in FxnKD cells. By pre-incubation with Vitamin E the abnormal effect induced by FxnKD was recovered during the response. However, the decay of the response was not completely restored.

We then looked at [Ca^2+^]_m_ after Thapsigargin administration and found that the mitochondrial re uptake was inefficient and slower in FxnKD than in controls (**Figure 8**). By pre-incubating Vitamin E, as an antioxidant, we found that we could recover cells with the calcium re-uptake. These results led us to conclude that the effect of oxidative stress is not only limited to the SR but also to the MCU uniporter involved in Ca^2+^ uptake (Dong et al., 2017). SR Ca^2+^ store depletion and associated contractile dysfunction are characteristics of systolic heart failure (HF) that are thought to be the consequence of depressed SERCA function, leaky RyRs and increased expression of NCX, mechanisms that might be implicated in the cellular pathogenesis of FRDA (Ying et al., 2008).

**FIGURE 9:**
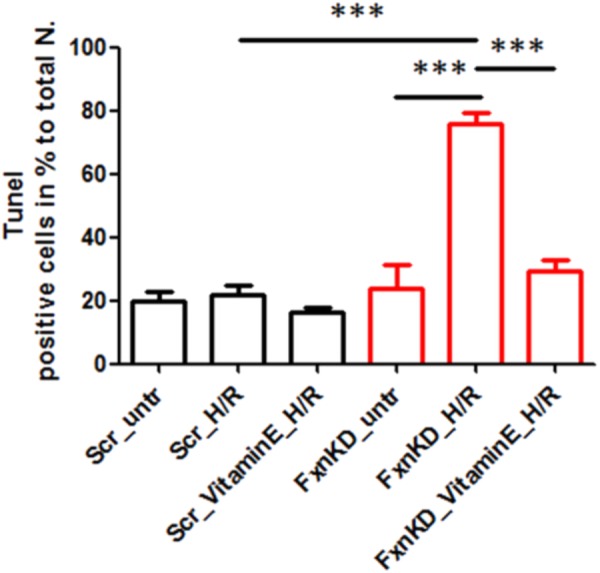
Vitamin E prevents cell death after hypoxia reperfusion. FxnKD cardiomyocytes exposed to H/R revealed an enounced vulnerability as the cell death increased significantly. However, cells pre-treated with Vitamin E seemed to be protected. The numbers of TUNEL-positive cells were calculated and the apoptotic cell death was expressed as percentage (^∗∗∗^*p* < 0.0005).

To this point we have challenged our cardiomyocytes with hypoxia reperfusion (H/R) to stress the cells and cause cell death. By investigating whether the H/R was pushing further the FRDA compromised phenotype or not we have also wondered if Vitamin E could protect the cells from apoptosis looking at TUNEL experiments. The results showed that apoptosis was increased in FxnKD cells compared to Scr and that could be prevented by Vitamin E administration (**Figure 9**, *Scr_untr* 19.7 ± 41, *Scr_H/R* 21.6 ± 5, *Scr_VitaminE_H/R* 16.52 ± 1.6; *FxnKD_untr* 23.7 ± 13, *FxnKD_H/R* 76 ± 6, *FxnKD_VitaminE_H/R* 29 ± 6.4; ^∗∗∗^*p* < 0.0005).

## Discussion

The reduced production of frataxin induces oxidative stress as a side effect of iron free radicals generation and mitochondrial energy imbalance (Puccio and Koenig, 2000; Bradley et al., 2004; Payne, 2011; Abeti et al., 2016). Neuronal and Cardiac tissues have high energy demand, and as such they are two of the target tissues where the function of frataxin is relevant (Puccio and Koenig, 2000). Ca^2+^ mishandeling in neuronal tissue can affect the capability to clear up the Ca^2+^ rise after action potential and impairing the post synaptic activity. Indeed, during an action potential Ca^2+^ rises by entering the cells via Voltage gated Ca^2+^ channels (VGCCs) and Ligand gated Ca^2+^ channels (LGCCs) and/or via its release from the ER. Then Ca^2+^ should be taken up by the internal stores and the homeostasis restored. By challenging CGNs with a depolarising insult, FRDA-like neurons could not restore their resting potential, leaving the membrane depolarised until death. This could be ascribed to a possible hyperactivity of either Inositol trisphosphate receptor (IP3Rs) or RyRs but also from an inhibition of the SERCA pumps impeding the Ca^2+^ to enter the ER and restore its cytosolic homeostasis. At the same time Ca^2+^ homeostasis in cardiac myocytes results from the integrated function of transsarcolemmal influx and efflux pathways modulated by membrane potential and from intracellular Ca^2+^ uptake and release predominantly by SR. Thus, modification of Ca^2+^ homeostasis may cause significant alterations in contraction and relaxation of the heart and cardiac failure (Limbu et al., 2015). Therefore, we focused our studies on Ca^2+^ homeostasis in cardiac FRDA like models. We showed that FxnKD in cardiomyocytes raised the level of ROS in the cytosol and in the mitochondria. We then demonstrated that Ca^2+^ homeostasis was altered, by challenging the cells with Caffeine and Thapsigargin, as the SR Ca^2+^ was lower in FxnKD cells compared to controls. The detrimental effect of Ca^2+^ signaling could be explained by the exacerbation of the oxidative stress in the FRDA like cells, since RyRs and SERCA present cysteines are affected by the variation of the oxidative environment (Marx et al., 2000; Diaz et al., 2005). Perhaps both RyRs and SERCA or one of the two could be altered. A reduction in Ca^2+^ content in the SR under pathological heart conditions is exacerbated by leaky Ca^2+^ release from the SR. It is known that hyperphosphorylation of the RyR2 (the predominant isoform in heart) increases its open probability (Ying et al., 2008) and also oxidative stress takes place in potentiating the channel. However, we cannot exclude a SERCA2a (the predominant isoform in heart) defect in pumping Ca^2+^ in the SR, as its reduction is strictly correlated to heart failure (Diaz et al., 2005).

SR Ca^2+^ store depletion and associated contractile dysfunction are characteristics of systolic heart failure (HF) that are thought to be the consequence of depressed SERCA function, leaky RyRs and increased expression of NCX, and this could be also the mechanism involved in FRDA (Ying et al., 2008).

On the other side we have observed that mitochondrial Ca^2+^ ([Ca^2+^]_m_) uptake is partially inhibited. This could be due to the effect of oxidative stress, not only limited to the SR, but also to the mitochondria and in particular to the mitochondrial calcium uniporter 1 (MCU) involved in [Ca^2+^]_m_ uptake (Dong et al., 2017). This was prevented by Vitamin E administration, indicating that oxidative stress is one of the major causes of Ca^2+^ deregulation in this model. Cells were then challenged with hypoxia/reperfusion (H/R) to induce cell death, and we have found that Vitamin E was protective for FxnKD cells. Vitamin E has been proposed as possible treatment for FRDA and cardiac abnormalities several years ago but never followed up due to lack of natural history on FRDA patients with this secondary effect to the pathology (Cooper and Schapira, 2007). Vitamin E, together with a cocktail of other vitamins, was proven to be protective in chronic HF (Witte et al., 2005). Patients who present with cardiomyopathy are often treated with bisoprolol to block the beta adrenergic receptors activated by the sympathetic nervous system (SNS) (Ramay et al., 2010). In physiological conditions the SNS is in resting state with no influence on heart function, but when heart failure occurs it becomes activated to maintain cardiac function by increasing the ionotropic support (including Ca^2+^) and oxidative stress potentiating RyRs (Ramay et al., 2010). Oxidative stress and RyRs potentiation is exactly what we have observed in our FRDA models. However, to preserve the cardiac muscle from oxidative stress and RyRs potentiation, an antioxidant able to protect the cells from death is needed. Ca^2+^ homeostasis has been studied in other FRDA cellular models such as neuronal cell lines and DRG, where the authors found Ca^2+^ homeostasis alteration (Bolinches-Amoros et al., 2014; Mincheva-Tasheva et al., 2014). In cardiomyocytes the RyRs are more important than others for the SR Ca^2+^ homeostasis; those results could indirectly confirm that the major problem of Ca^2+^ homeostasis disruption in our cardiac FRDA-like models is generated by defective RyRs (Bolinches-Amoros et al., 2014; Mincheva-Tasheva et al., 2014).

## Conclusion

Our findings have shown a decreased ER/SR Ca^2+^ content in FRDA-like neurons and cardiomyocytes. In neurons this could be due to SERCA pumps inhibition which needs to be further investigated. Although a similar phenomenon could happen also in cardiomyocytes, we have proven that there could be a problem perhaps because of RyRs potentiation bringing out a greater portion of Ca^2+^ from the SR. The effect of dantrolene, an inhibitor of RyRs, proved that the receptors were not inhibited as the SR Ca^2+^ was nearly restored. Mitochondrial Ca^2+^ uptake was found inhibited, similarly to other FRDA-like models (Bolinches-Amoros et al., 2014) and Vitamin E restored the signal and prevent from apoptosis induced by H/R. Mitochondrial dysfunction present in FxnKD cardiomyocytes, was also found to be strictly related to atrial dilation (Roosimaa et al., 2013) in other disorders that develop hypertrophic cardiomyopathy. Our study opens potential trials for cardiomyopathy in FRDA but also reinforces the need to use antioxidants in conventional HF where similar mechanisms operate. This work shed a light on how the modulation of Ca^2+^ in FRDA-like cardiomyocytes could be exploited to provide novel therapeutic strategies.

## Author Contributions

RA designed the study, conducted and designed the experiments, analyzed the results, and wrote the manuscript. AB contributed to performing the experiments. MM contributed to performing the experiments and interpreting the data and editing the manuscript. SP contributed to interpreting the data and editing the manuscript. PG contributed to design the study and to write the manuscript.

## Conflict of Interest Statement

The authors declare that the research was conducted in the absence of any commercial or financial relationships that could be construed as a potential conflict of interest.

## Supplementary Material

FIGURE S1 Frataxin KD in cardiomyocytes. **(A)** HL-1 cells transfected with Scr and FxnKD. From top left Scr YFP cells (green), and labeled with Fxn antibody, showing red fluorescence. Bottom left, DAPI to label the nuclei (blue) and merge. On the top right panel YFP positive cells in FxnKD and labeled with Fxn antibody, showing a very low red fluorescence due to the KD. Bottom right, DAPI to label the nuclei (blue) and merge. **(B)** The histogram shows the quantification of the Fxn red fluorescence detected in the dishes and comparing Scr and FxnKD. FxnKD showed a significant decrease of the Fxn level (^∗^*p* < 0.05). Scale bars (20 μm). **(C)** H9c2 cells transfected with scr and FxnKD. From top left Scr YFP cells (green), and labeled with Fxn antibody, showing red fluorescence. Bottom left, DAPI to label the nuclei (blue) and merge. On the top right panel YFP positive cells in FxnKD and labeled with Fxn antibody, showing a very low red fluorescence due to the KD. Bottom right, DAPI to label the nuclei (blue) and merge. **(D)** The histogram shows the quantification of the Fxn red fluorescence detected in the dishes and comparing Scr and FxnKD. FxnKD showed a significant decrease of the Fxn level (^∗∗^*p* < 0.005). Scale bars (20 μm).

## Abbreviations

Ca^2+^: calcium
CGNs: cerebellar granule neurons
FRDA: Friedreich’s ataxia
ROS: reactive oxygen species
RyRs: ryanodine receptors
SERCA: sarco/endoplasmic reticulum Ca^2+^-ATPase
SR: sarcoplasm reticulum
