# Supplementary figures and images for: Calcium Deregulation: Novel Insights to Understand Friedreich’s Ataxia Pathophysiology
Source: Front Cell Neurosci. 2018 Oct 2;12:264. doi: 10.3389/fncel.2018.00264 (PMC6176067; doi:10.3389/fncel.2018.00264)

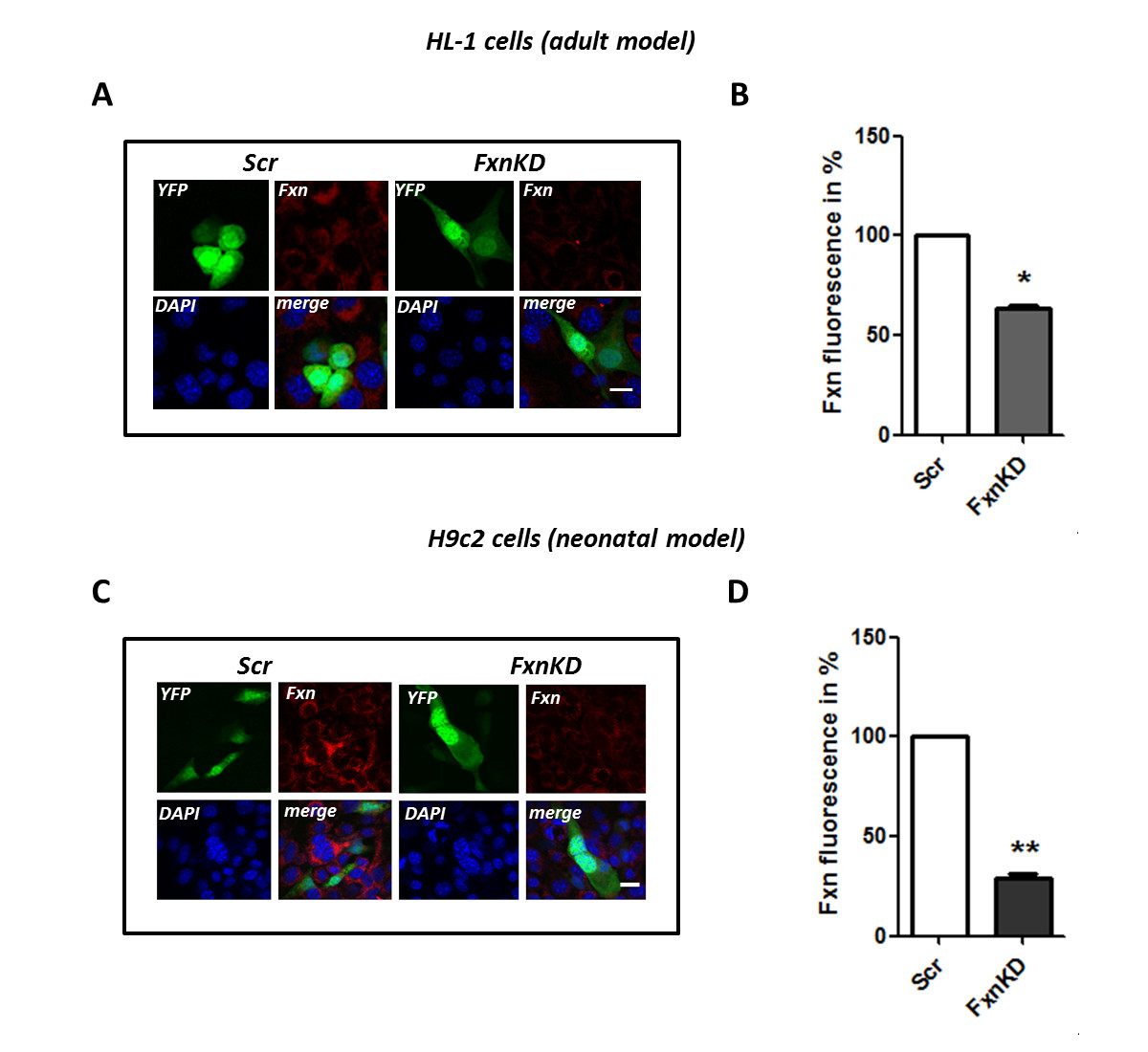

Supplement: FIGURE S1 — Frataxin KD in cardiomyocytes. (A) HL-1 cells transfected with Scr and FxnKD. From top left Scr YFP cells (green), and labeled with Fxn antibody, showing red fluorescence. Bottom left, DAPI to label the nuclei (blue) and merge. On the top right panel YFP positive cells in FxnKD and labeled with Fxn antibody, showing a very low red fluorescence due to the KD. Bottom right, DAPI to label the nuclei (blue) and merge. (B) The histogram shows the quantification of the Fxn red fluorescence detected in the dishes and comparing Scr and FxnKD. FxnKD showed a significant decrease of the Fxn level (∗p < 0.05). Scale bars (20 μm). (C) H9c2 cells transfected with scr and FxnKD. From top left Scr YFP cells (green), and labeled with Fxn antibody, showing red fluorescence. Bottom left, DAPI to label the nuclei (blue) and merge. On the top right panel YFP positive cells in FxnKD and labeled with Fxn antibody, showing a very low red fluorescence due to the KD. Bottom right, DAPI to label the nuclei (blue) and merge. (D) The histogram shows the quantification of the Fxn red fluorescence detected in the dishes and comparing Scr and FxnKD. FxnKD showed a significant decrease of the Fxn level (∗∗p < 0.005). Scale bars (20 μm). [file Image_1.jpg]
